# Polymorphic Variants of *FOXP3* Gene (rs 3761548) and (rs 3761549) are Significantly Associated with the Risk for Recurrent Pregnancy Losses. A Study in High Incidence Zone (Kashmir, North India)

**DOI:** 10.34763/jmotherandchild.20252901.d-25-00030

**Published:** 2025-12-04

**Authors:** Usma Manzoor, Arshad A. Pandith, Tawseef A. Lone, Amreena Hameed, Falak U Nisa, Ina Amin, Shayesta Rah, Saima Wani, Shayaq Ul Abeer Rasool, Adil Lateef, Aabida Ahmad

**Affiliations:** Advanced Centre for Human Genetics, Sher-I-Kashmir Institute of Medical Sciences (SKIMS), Srinagar-190011, J&K-India; Department of General Surgery, Sher-I-Kashmir Institute of Medical Sciences (SKIMS), Srinagar-190011, J&K-India; Department of Clinical Biochemistry, University of Kashmir-190006,J&K-India; Department of Obstetrics and Gynecology, Government Associated (LD Hospital), Srinagar-190001, J&K, India; Department of Obstetrics and Gynecology, Sher-I-Kashmir Institute of Medical Sciences (SKIMS), Srinagar-190011, J&K-India

**Keywords:** FOXP3, Recurrent Pregnancy Loss, Polymorphic Variations, Treg cells, Genotyping

## Abstract

**Objective:**

Recurrent Pregnancy Loss (RPL) is a significant pregnancy complication with a multifactorial aetiology and is a vital reproductive health concern that globally affects 2–5% of women. Polymorphic gene variation causes alteration in FOXP3 gene that impairs the Treg cells which leads to complications in pregnancy outcome. Thus, we aimed to study an association between FOXP3 polymorphic variations (rs3761548 and rs3761549) and RPL.

**Material and methods:**

This case control study comprised of 120 RPL cases and 150 healthy multiparous women as control group with at least one full term pregnancy and no history of pregnancy loss matched to cases according to age and geographic origins. Genotyping for FOXP3 was analyzed by polymerase chain reaction - restriction fragment length polymorphism (PCR-RFLP).

**Results:**

Significantly higher frequency of FOXP3 −3279 C/A (rs 3761548)heterozygous AC and homozygous AA was found in RPL cases than controls (63.3% vs. 46%, O.R = 2.53): p = 0.0006 and (11.7% vs. 8%; O.R 2.68): p = 0.03 respectively. Moreover, the dominant model (AC +AA) and allele A were seen implicated more in RPL cases vs. healthy control (75% vs. 54%; O.R = 2.5): p = 0.0005 and (43.3% vs. 31%; O.R = 1.7): p = 0.003. For FOXP3 −2383 C/T (rs 3761548), homozygous genotype TT was significantly higher in RPL cases than the control group against the wild type CC genotype with O. R= 3.49 (p = 0.04). Further, FOXP3 (rs 3761548) genotypes AC+AA were significantly associated between cases and control in terms of women without any known family history (p = 0.0009) and consanguinity (p = 0.0002), respectively.

**Conclusion:**

The study concludes that both the variants of FOXP3 gene, C/A (−3279) and C/T (−2383) are significantly associated with an increased risk for recurrent pregnancy losses.

## Introduction

Recurrent Pregnancy Loss (RPL), presently defined as the failure of two or more clinically recognised pregnancies before the 20^th^ week of gestation [1]. It is a significant pregnancy complication affecting 1–3% of couples attempting to conceive [2]. RPL is a multifactorial, although many known causes were shown to contribute to its pathogenesis, including anatomic, infectious, hormonal, immunological and genetic predisposing aspects, while around 40–50 % of the causes remain idiopathic [3]. It is worth mentioning that present studies have indicated the role of miRNAs in the regulation of genes involved in the pivotal processes for the maintenance of a successful pregnancy outcome [4,5]. In addition, previous reports have suggested that RPL is associated with the failure of foetal-maternal immunologic tolerance, and disturbed immune response has been suggested as a pivotal mechanism to understand the underlying aetiology [6].

Regulatory T cells (Tregs) play a crucial role in the induction and development of an immune-tolerant environment at the foetal-maternal interface, thus supporting the maintenance of pregnancy [7]. It has been found that RPL patients have reduced rates of CD4+CD25+Treg cells in peripheral blood as well as in decidua [8], which is strongly associated with reduced expression of the *FOXP3* gene [8]. *FOXP3* plays a key role in the development and functioning of Treg cells, while decreased expression of the *FOXP3* gene impairs the suppressive function of Treg cells by affecting the development of CD4+CD25+ Tregs [9]. This reduction in Tregs (CD4+CD25+) in RPL patients is closely linked to the immunological barrier disruption at the foetal-maternal interface [8]. The human *FOXP3* gene is positioned on chromosome X(Xp11.23)-a member of the Forkhead/winged-helix family, and is also known as “Scurfin”, which encodes a transcription factor required for the formation and activity of CD4+CD25+Tregs [10]. Previous reports have found polymorphisms in the promoter region of *FOXP3* gene affect transcription initiation and thus, gene expression. Furthermore, autoimmune diseases like systemic lupus erythematosus (SLE), autoimmune thyroid diseases (AITDs) [11], and type I diabetes (TID) [12] have been linked to polymorphisms in the *FOXP3* gene. Studies in different populations have also observed the association between RPL and *FOXP3* gene polymorphisms, including the Intron 1 rs2232365 (− 924 G > A), rs3761548 (− 3279C > A), and rs5902434 (− 6054) and rs2294021 [14–13]. In view of the plausible role between *FOXP3* gene and RPL, this study was carried out for the first time in the Kashmiri population to assess the association of the *FOXP3* genetic variants and RPL to analyse the difference in genotype and allele frequencies among cases and controls to rule out the any possible association and risk thereof.

## Material and Methods

### Participants

In this retrospective case-control study conducted in Advanced Centre for Human Genetics, SK Institute of Medical Sciences (SKIMS), Srinagar (J&K, India), cases comprised of 120 Kashmiri women of reproductive age group diagnosed with RPL attending the Department of Gynecology and Obstetrics, SKIMS. The inclusion criteria for RPL patients were at least 2 pregnancy losses in ≤ 20 weeks of gestation. The mean age of RPL patients was 29.83 ± 4.47 years. Exclusion criteria included patients with chromosomal aberrations assessed by karyotyping, uterine anatomical abnormalities, infections, and patients with immune and endocrine disorders. The control group included 150 healthy multiparous women matched to cases according to age (mean age 29.58±3.98 years), ethnicity and geographic origins having at least one full term pregnancy and no pregnancy loss in obstetric history. A written pre-informed consent was obtained from all cases and controls. Clinico-pathological features of each subject were documented in a questionnaire. Informed consent was obtained from all participants after approval of the study by the Institutional Ethical Committee (SKIMS Study ref: Protocol RP 244/2019).

### Sample collection and DNA Extraction

A total of 3ml peripheral venous blood samples were collected from all the participants in EDTA-vials for total genomic DNA extraction using a QAIGEN (Hilden, Germany) DNA extraction kit method as per the manufacturer’s protocol. The isolated DNA was stored at −20°C till further investigation of *FOXP3* gene polymorphism.

### Genotyping of *FOXP3* polymorphism

**Figure 1.**
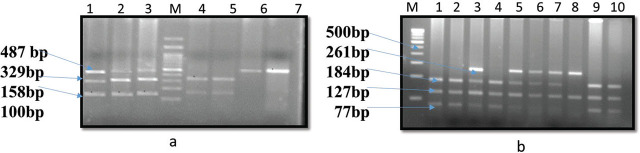
Representative gel picture showing restriction digestion of *FOXP3* −3279 (rs 3761548) (a) and −2383 (rs 3761549) (b) for genotyping (a) L1-L3: Heterozygous (487,329,158bp), L4 and L5: Homozygous wild (329,158bp), L6 and L7: Homozygous variant (487bp), M=100bp. (b) L1, L2, L4, L9, L10: Homozygous (184,127,77 bp), L3, L5, L6, L7: Heterozygous (261,184,127,77 bp), L8: Homozygous variant (261, 127bp), M= 100bp. bp=base pair, M=marker.

Genetic polymorphism for *FOXP3*-3279 C/A (rs 3761548) and −2383 C/T (rs 3761549) was analysed by polymerase chain reaction - restriction fragment length polymorphism (PCR-RFLP) protocol using the primers for *FOXP3* −3279 C/A as forward primer-F-5′-GCTCTACTTCCTGAAGACCT-3´ and reverse primer R-5′-AGTCTCACTCACCTTT GCAG-3′ and for *FOXP3* 2383C/T-forward primer F-5′-AAATGAATTGGACTGGATGGT-3′ and reverse primer-R-5′-TTACGAGAAAGGAAGCCGTG-3´. Annealing temperature for both SNPS was 63°C and 65°C, yielding amplicon sizes of 487bp and 261bp, respectively. An aliquot of 10ul PCR products were digested with 1ul of restriction enzyme *PstI* (New England, Biolabs UK) at 37°C on duration of 16hr for −3279 C/A and 1ul of *BseNI* (New England, Biolabs UK) at 65°C on duration of 16hr for −2383 C/T and then separated on 3% agarose gel as represented in **Figure 1.**

### Statistical Analysis

The genotype, allele frequency in RPL patients and the controls were analysed by the standard chi-square test and odds ratio (OR) for risk of RPL at 95% confidence intervals (CI). Statistical analyses were performed using the SPSS 17.0 software package (SPSS, Chicago, I/l, USA) and Graph Pad Prism 8.0.2. Statistical significance was considered at *p* < 0.05. To account for multiple comparisons, we applied the Bonferroni correction. We found that our main findings remained statistically significant even after applying the correction for multiple comparisons.

## Results

The cases and controls were frequency matched in terms of their age and dwelling. The mean age of RPL cases and controls were 29.83 ± 4.47 and 29.58 ± 3.98 years respectively. The clinico-pathological characteristics of study subjects are shown in **Table 1**. In addition to that, the number of live births was found to be significantly lower in women with RPL as compared to the control population (*p* < 0.0001). Family history (*p* = 0.03) and consanguinity (*p* = 0.01) were also significantly related between cases and the control group. Age, body mass, smoking and irregularity in menstrual functions were comparable between RPL cases and control women.

**Table 1.** Demographic and clinical characteristics of study RPL and Control participants.

**Clinico-pathological characteristics**	**Cases Mean ± SD**	**Control Mean ± SD**	**Chi Square Test p value**
**Age**	29.83 ± 4.47	29.58 ± 3.98	0.690
**Live Birth**	0.40 ± 0.66	2.38 ± 0.62	<0.0001
**Number of Pregnancies**	3.35 ± 1.14	2.65 ± 0.82	<0.0003
**Miscarriages**	2.81 ± 1.08	0.00 ± 0.00	-
**Women with no live birth**	44 (36%)	0	-
**Family History**			
**YES**	23 (19.1%)	15 (10%)	Ref
**NO**	97 (80.8%)	135 (90%)	0.03
**Consanguinity**			
**YES**	38 (31.6%)	27 (18%)	Ref
**NO**	82 (68.4%)	123 (82%)	0.01

*p* value was calculated by Chi Square Test.

### Association studies

The distribution of *FOXP3* −3279 C/A (rs 3761548) and −2383 C/T (rs 3761549) genotypes was in Hardy-Weinberg equilibrium among the control population. **Table 2**shows the distribution of genotypes for *FOXP3* polymorphism in study subjects. *FOXP3* −3279 C/A frequencies of CC, CA, and AA genotype among cases were 30 (25%), 76 (63.3%) and 14 (11.7%), and 69 (46%), 69 (46%), and 12 (8%) respectively in the controls. The frequencies of the CC, CT, and TT genotypes of 2383 C/T in the RPL women were 68 (56.6%), 42 (35%) and 10 (8.3%), while as in controls it was 95 (63.4%), 51 (34%) and 4 (2.6%) respectively. Two different models were used for this study, additive and dominant model as shown in **Table 2.**These two models were found fit for this study to assess the genotype and allelic distribution among cases and controls. The frequency of heterozygous AC and homozygous AA for rs 3761548 was found significantly higher in cases with RPL than controls (63.3% vs. 46%) O.R = 2.53: *p* = 0.0006 and (11.7% vs. 8%) O.R 2.68: *p* = 0.03 respectively. Moreover, the dominant model (AC +AA) and the A allele were significantly increased in cases as compared to healthy controls (75% vs. 54%) O.R = 2.5: *p* = 0.0005 and (43.3% vs. 31%) O.R = 1.7: *p* = 0.003 (**Table 2**).

**Table 2.** Genotypic and allelic frequencies of *FOXP3* 3279 C/A and 2383 C/T polymorphism in RPL cases and controls.

**SNP**	**Genotype/Allele**	**Cases (%) N=120**	**Controls (%) N=150**	**OR (95% C.I)**	***p* value**
**FOXp3-3279 C/A**	CC	30 (25)	69 (46)	Ref	Ref
	AC	76 (63.3)	69 (46)	2.53 (1.47–4.34)	0.0006
	AA	14 (11.7)	12 (8)	2.68 (1.1–6.4)	0.03
	AC+AA	90 (75)	81 (54)	2.5 (1.5–4.3)	0.0005

**Alleles**	C	136 (56.6)	207 (69)	Ref	Ref
	A	104 (43.3)	93 (31)	1.70 (1.1–2.4)	0.003

**FOXp3-2383 C/T**	CC	68 (56.6)	95 (63.4)	Ref	Ref
	CT	42 (35)	51 (34)	1.15 (0.6–1.9)	0.60
	TT	10 (8.3)	04 (2.6)	3.49 (1.0–11.6)	0.04
	CT+TT	52 (43.3)	55 (36.6)	1.32 (0.8–2.1)	0.31

**Alleles**	C	178 (74.2)	241 (80.3)	Ref	Ref
	T	62 (25.8)	59 (19.7)	1.42 (0.9–2.1)	0.09

Fishers Exact Test: *p* value < 0.05 is considered statistically significant; O.R: Odds Ratio: 95% C.I: .95 Confidence Intervals; Ref: Reference; SNP: Single Nucleotide Polymorphism.

For *FOXP3*-2383 C/T polymorphism, RPL cases were carrying significantly higher homozygous genotype TT in cases than in the controls with O. R = 3.49 (*p* = 0.04). However, no significant differences in the CT heterozygous frequency were noted between RPL and controls (O.R =1.15: *p* = 0.60). Regarding the T allelic distribution as shown in **Table 2**, RPL cases with T allele showed borderline statistical significance against the control population with O.R = 1.42: *p* = 0.09.

On stratification with respect to different risk parameters, patients with RPL who had <30 years of age were distinctly associated with combined variant genotype AC+AA of rs3761548 C/A and CA+TT for rs 3761549 C/T denoting (O.R = 3.1; *p* = 0.001) and (O.R = 2.0; *p* = 0.04) respectively. The distribution of genotypes AC+AA for rs3761548 were significantly associated between cases and control in terms of women without any known family history (*p* = 0.0009) and consanguinity (*p* = 0.0002) respectively (**Table 3**).

**Table 3.** Combined genotypes of *FOXP3* −3279 C/A and −2383 C/T and risk of RPL with respect to clinical characteristics.

**Marker**	**Parameter**		**Cases n=120**		**Controls (N=150)**		**O.R (95%CI)**	**Fishers Exact Test *p* value**

			**CC**	**AC+AA**	**CC**	**AC+AA**		
**rs3761548**	Age	≤30 years	20 (16.6%)	54 (45%)	39 (26%)	33 (22%)	3.1 (1.5–6.3)	**0.001**
		>30 years	10 (8.3%)	36 (30%)	30 (20%)	48 (32%)	2.2 (0.9–5.1)	0.07
	F/H	Yes	5 (4.1%)	18 (15%)	5 (3.3%)	10 (6.6%)	1.8 (0.4–7.7)	0.47
		No	25 (20.8)	72 (60%)	64 (42.6%)	71 (47.3%)	2.5 (1.4–4.5)	**0.0009**
	Cons.	CM	9 (7.5%)	29 (24.1%)	6 (4%)	21 (14%)	0.9 (0.2–3.0)	1
		NCM	21 (17.5%)	61 (50.8%)	63 (42%)	60 (40%)	3.0 (1.6–5.6)	**0.0002**

**rs3761549**			CC	CC+TT	CC	CC+ TT		

	Age	≤30 years	40 (33.3%)	34 (28.3%)	51 (34%)	21 (14%)	2.0 (1.0–4.0)	**0.04**
		>30 years	28 (23.3%)	18 (15%)	44 (29.3%)	34 (22.6%)	0.8 (0.3–1.7)	0.7
	F/H	Yes	11 (9.16%)	12 (10%)	8 (5.3%)	7 (4.6%)	1.2 (0.3–4.5)	0.9
		No	57 (47.5%)	40 (33.3%)	87 (58%)	48 (32%)	1.2 (0.7–2.1)	0.4
	Cons.	CM	18 (15%)	20 (16.6%)	17 (11.3%)	10 (6.6%)	1.8 (0.6–5.1)	0.3
		NCM	50 (41.6%)	32 (26.6%)	78 (52%)	45 (30%)	1.1 (0.6–1.9)	0.7

*P* value < 0.05 is considered statistically significant; O.R: Odds Ratio; 95% C.I: .95 Confidence Intervals; F/H: Family History; Cons: Consanguinity; NCM: Non-Consanguineous Marriage.

## Discussion

For the successful implantation and maintenance of the fetus, Treg cells play a vital role in foetal-maternal immunologic tolerance. It is evident that the *FOXP3* controls the successful development of Treg cells, which are believed to be one of the most prominent immunological factors investigated in RPL [15]. Dysregulated immune response was considered as a potential underlying cause for RPL [16,17] that substantiates its crucial role to regulate immune response to support a favourable pregnancy outcome [18]. There are evidences which show defective number of Tregs (CD4+,CD 25+) in peripheral blood as well as the decidua of RPL patients and its association with decreased expression of *FOXP3* in the women with poor pregnancy outcome [8]. Polymorphisms in the promotor region of the *FOXP3* gene could result in altered gene expression by affecting the initiation of transcription caused by inefficient binding of transcription factor and thus leads to immune system imbalance and mediate clinically serious human disease development like recurrent abortions.

In this study, we observe that among Kashmiri women population, the frequency of variant genotype AA in *FOXP3*- 3279 C/A (rs 3761548) was significantly higher in cases than control population (0.11% vs. 0.8%: p < 0.05) and clearly demonstrates a significant association of *FOXP3* −3279 C/A genotype with the susceptibility of subjects to develop the risk for RPL in our population. Our findings agree with the study by Jaber et al. [19], where AA genotype and the A allele frequency were significantly higher in RPL women (OR = 2.86 and OR = 1.75) respectively. The results of our study were also in accordance to the findings of Wu et al. [20] and Sharif et al. [21] that reported a substantial contribution of *FOXP3* −3279 C/A gene polymorphism with recurrent pregnancy losses in different countries of Asian ethnicity. Our findings were further corroborated by the observations of Saxena et al. [14] and Dirsipam et al. [22] in the Indian population, wherein the hazard of RPL was indicated as 2- 3 fold in patients with polymorphic variants of SNP rs3761548 C/A in the *FOXP3* gene. A meta-analysis done by Bamba et al. [23] observed SNP rs3761548 C/A as the most common variant among various Asian countries. Thus, our data provided more evidence in supporting the predisposing role of *FOXP3* polymorphism in RPL. On the contrary, a previous study by Hadinedoshan et al. [24] on the Iranian population concluded unavailing results for *FOXP3* –3267 C/A (rs3761548) polymorphisms in women with RPL. In addition, the investigation done by Mishra et al. [25] for rs3761548 C/A was also in disagreement with the earlier studies. In this study for rs3761548, the observed frequency of the C allele was 56.6% and 43.3% for A allele in cases vs. 69% and 31% in controls respectively. In line with our study, Zaigui et al. [20] found the frequency of C and A alleles as 29.45% vs. 42.40% and 70.55% vs. 57.60% in cases and controls, respectively (*p* = 0.003). Thus, polymorphic variations in *FOXP3* gene may result in the dysregulated expression of *FOXP3* and significantly impact the modulation of suppressive capabilities of Tregs [26]. The abated function of regulatory T cells, in turn, plays a key role in the abnormal expression of various immune cells, causing an unfavourable pregnancy outcome. Our study revealed a greater proportion of variant alleles in the age group ≤ 30 years in cases than control group (45% vs 22%: *p* = 0.001). Furthermore, we observed significant association of minor allele A in patients without any family history and non-consanguinity as compared to control population (*p* < 0.05). As there is a limited number of investigations regarding this subject, the discussed parameters need to be studied further in detail to come up with more authentic results.

For *FOXP3* gene, rs3761549 C/T, genotypic and allelic distribution portrayed more than 3-fold elevated risk of RPL in women carrying TT genotype than CC genotype (*p* = 0.04) but showing borderline significance for T allele between the two groups (cases vs. controls: **Table 2**). However, it is worth mentioning that the presence of T allele has been observed to be significantly higher in patients with lung carcinoma, thus suggesting its role in susceptibility to the disease [27]. There is ample evidence for suggesting the association between *FOXP3-*2383 C/T (rs3761549) polymorphism and some autoimmune diseases. One of the earlier studies has elucidated a genetic association between the FOXP3 gene and Psoriasis in the Chinese majority population, suggesting the T allele as a contributing factor to the disease [28]. Furthermore, Lan et al. [29] attributed the association between *FOXP3*-2383 SNP and genetic susceptibility to systematic lupus erythematosus in Guangxi Zhuang population signifying relative risk of suffering as 1.71 times in T allele carriers (p <0.05). Interestingly, these results are also consistent with the other studies done by Andre et al. [30] for single marker analysis of *FOXP3* polymorphisms (rs3761549) revealing a possible association with endometriosis in Brazilian population (P= 0.03). Nevertheless, stratification of genotypic and allelic analysis of this SNP have failed to detect any implication in hepatocellular carcinoma and breast carcinoma [31] in Asian ethnicity. *FOXP3* rs3761549 polymorphisms have not previously been investigated in women with RPL. This is the first prospective study in this region to actively follow women for recurrent abortions. Our study has demonstrated the possible role of TT genotype in *FOXP3*-2383 C/T SNP indicating immunopathogenesis in patients with RPL. Overall keeping in view the importance of this gene, many studies across the globe that explored the association between *FOXP3* variants and RPL came upon inconclusive and inconsistent but interesting results and our study has further strengthened its importance (**Table 4**). Thus, further study of *FOXP3* polymorphic variations may contribute to a better understanding of their implications in conferring risk to RPL.

**Table 4.** Results of analysis for the selected SNPs with RPL risk and other diseases.

**S. No.**	**Study**	**Country/Ethnicity**	**Case/Control**	**Disease**	***p* value**
	**3279 FOXP3 rs3761548**				

**1.**	Naderi et al., 2015 [13]	Iran/Asian	195/101	RPL	0.387
**2.**	Saxena et al., 2015 [14]	India/Asian	200/300	RPL	**<0.001**
**3.**	Jabar et al., 2014 [19]	Palestine/Asian	100/100	RPL	**0.005**
**4.**	Wu et al., 2012 [20]	China/Asian	146/112	RPL	**0.003**
**5.**	Sharif et al., 2016 [21]	Palestine/Asian	100/100	RPL	**0.011**
**6.**	Dirsipam et al., 2021 [22]	India/Asian	150/150	RPL	**<0.001**
**7.**	Hadinedoshan et al., 2015 [24]	Iran/Asian	80/80	RPL	0.30
**8.**	Mishra et al., 2018 [25]	India/Asian	100/100	RPL	0.18
**9.**	Our study (2024)	India/Asian	120/150	RPL	**<0.001**

	**2383 Foxp3 rs3761549**				

**1.**	Song et al., 2012 [27]	China/Asian	408/363	Psoriasis vulgaris	**<0.05**
**2.**	Lan et al., 2010 [28]	China/Asian	120/160	SLE	**<0.05**
**3.**	Andre et al., 2011 [29]	Brazil/SA	177/171	Endometriosis	**0.003**
**4.**	Tian et al., 2018 [30]	China/Asian	560/582	Breast Cancer	n. s
**5.**	Our study (2024)	India/Asian	120/150	RPL	**0.04**

SLE: Systematic lupus erythematosus, RPL: Recurrent Pregnancy Loss.

Limitations of the study include a slightly smaller sample size, keeping in view the high incidence of RPL in our region. Besides, study augments for the expression analysis of *FOXP3* gene and its association with genotypes. Future studies that incorporate longitudinal *FOXP3* protein levels, functional genomics, and epigenetic modifications would be pivotal in demonstrating a more reliable knowledge of FOXP3-mediated role in etiopathogenesis of RPL. In addition, further studies are required with the inclusion of more *FOXP3* polymorphic variations to analyse the linkage disequilibrium. Furthermore, the data of the study underscores the need for different ethnic based studies to confirm significant association of the genetic variants with respect to the RPL.

## Conclusion

The study concludes that *FOXP3* gene, C/A (−3279) and C/T (−2383) are significantly associated with an increased risk for RPL. Also, the exhibition of *FOXP3* variants infers a more pronounced risk in the lower age group. Our data suggests a need to authenticate the results through expressional epigenetic regulation and functional validation studies in a series of cohort studies to confirm its possible implications for RPL.
